# Augmented Analytics Driven by AI: A Digital Transformation beyond Business Intelligence

**DOI:** 10.3390/s22208071

**Published:** 2022-10-21

**Authors:** Noorah A. Alghamdi, Heyam H. Al-Baity

**Affiliations:** 1 Management Information Systems Department, College of Business Administration, King Saud University, Riyadh 11362, Saudi Arabia; 2 Information Technology Department, College of Computer and Information Sciences, King Saud University, Riyadh 11362, Saudi Arabia

**Keywords:** Artificial Intelligence, Augmented Analytics, Business Intelligence, citizen data scientists, Machine Learning, Natural Language Processing, Natural Language Generation

## Abstract

Lately, Augmented Analytics (AA) has increasingly been introduced as a tool for transforming data into valuable insights for decision-making, and it has gained attention as one of the most advanced methods to facilitate modern analytics for different types of users. AA can be defined as a combination of Business Intelligence (BI) and the advanced features of Artificial Intelligence (AI). With the massive growth in data diversity, the traditional approach to BI has become less useful and requires additional work to obtain timely results. However, the power of AA that uses AI can be leveraged in BI platforms with the use of Machine Learning (ML) and natural language comprehension to automate the cycle of business analytics. Despite the various benefits for businesses and end users in converting from BI to AA, research on this trend has been limited. This study presents a comparison of the capabilities of the traditional BI and its augmented version in the business analytics cycle. Our findings show that AA enhances analysis, reduces time, and supports data preparation, visualization, modelling, and generation of insights. However, AI-driven analytics cannot fully replace human decision-making, as most business problems cannot be solved purely by machines. Human interaction and perspectives are essential, and decision-makers still play an important role in sharing and operationalizing findings.

## 1. Introduction

Leveraging analytics as a key success factor empowers businesses to gain a competitive advantage in the market. With the increase in investments in digital transformation, data volumes have become huge, and manual approaches to performing analytics, discovering interesting data patterns, and coming up with useful insights for decisions are becoming increasingly challenging. Thus, advanced techniques for managing, analyzing, and visualizing big data have become critical and have gained considerable attention recently. Augmented Analytics (AA) is the next wave and the future trend of the data analytics market, and many businesses are investing in AA as early adopters to differentiate themselves and obtain more strategic business benefits [1].

AA encompasses the use of Machine Learning (ML), Artificial Intelligence (AI), Natural Language Processing (NLP), and Natural Language Generation (NLG) techniques to automate the analytics cycle for business and transform the ways in which analysts execute and share data insights. The AA approach facilitates data preparation, insight generation, and interpretation, and augments the ways in which users explore and analyze data in analytics and Business Intelligence (BI) platforms. In other words, it acts like a virtual data scientist who executes analysis activities and operationalizes data findings [2]. Thus, leveraging the full potential of AA features makes analytics accessible and easier to use, resulting in more data-driven and timely decision-making for overall organizational growth [3].

AA is expected to be a dominant driver of BI solutions and embedded analytics that businesses will look for whenever they need to scale their analytics operations [4]. AA allows data scientists to be more productive by extending the capabilities of Data Science (DS) and ML, and makes the data easier to use for less skilled users [5]. This leads to the introduction of new “citizen data scientists” who can perform advanced analytics along with predictive and prescriptive capabilities when statistics or analytics is not in the scope of their primary job function [6]. Furthermore, AA supports natural language and conversational interfaces that enable users to run text and voice queries [4]. Moreover, it provides business users with unbiased data analysis, highlights unknown growth opportunities, and supports agile enterprises [3]. In addition, it improves the utilization of resources, as data analysts can focus on the more difficult tasks that cannot be fully automated. AA enables business users to obtain rapid answers to their questions without the need to fully rely on data analysts to bridge the gap [7].

Over the years, BI has been used to perform reporting, visualization, and scorecards. It has been through several generations, where the first generation only had simple reporting features. Later, in the second generation, it was developed to introduce self-service BI, making users more capable of performing analysis tasks. However, in its most recent generation, BI has been transformed with the introduction of AI and AA functionalities. It is important to note that AA is only a new term in business analytics tasks, which fully corresponds to the current practices of AI, ML, and data mining. The AA includes preparing the data, finding interesting patterns in the data, and sharing and operationalizing the findings. In other words, AA uses ML, AI, NLP, and NLG to automate the analytics process for BI. For instance, during data preparation in the traditional BI platforms, various tasks are performed manually to find relationships between variables, augment data profiling, develop and catalogue metadata, and recommend preferable methods for cleaning, reconciling, enriching, and manipulating the data. AI in this case is used to suggest associations between data sources and to find patterns in the data. In addition, ML algorithms are used to automate the detection of correlations, links, associations, and outliers. The analyst may use NLP to ask questions and find answers in the form of visuals that may help in finding insights. Consequently, intelligent visualization and interactive dashboards with storytelling are used to share findings from the data, with the support of collaboration capabilities to show patterns and hidden insights [1,3].

This study mainly highlights the traditional approach of BI compared with AA features added to BI platforms, along with how AI is transforming BI by utilizing AA to keep pace with big data. In Section 2, generations of BI are identified with reasons for expanding from BI to AA, along with the process of using AA platforms in the business analytics cycle. Furthermore, related works that describe and summarize empirical articles are presented in Section 3 to explain the benefits of AA and the use of augmented BI platforms in different industries. Section 4 describes the methodology of the study. The results and analysis of the comparison between the capabilities of the two approaches are presented in Section 5 and discussed in Section 6. Finally, the conclusions and future prospects are presented in Section 7.

## 2. Background

The growth of data volume and the high competition in the market have made investing in intelligent tools a priority for businesses in gaining a competitive advantage. BI has been developed over the years, beginning as a query and reporting software module and continuously evolving with additional features such as Online Analytical Processing (OLAP), mobile BI, data visualization, and scorecards. BI software continues its transformation process with the introduction of AI and ML into its functionalities to automate data analysis. In addition, Deep Learning (DL) has begun to be considered as an added value for BI platforms in the market [8]. A new approach was introduced by Gartner in 2017 that incorporates BI and advanced analytics such as AA and is expected to be the future of big data and analytics [3].

AA is an emerging trend that utilizes AI, ML, NLP, and NLG technologies at all stages of the analytics cycle for BI, with a simplified process that helps analysts in their tasks. The main stages of the analytics cycle are the Ask, Consider, Analyze, and Interpret stages. AA is applied in the Ask stage by utilizing NLP and by allowing users to ask questions using their voice or typed text. In the Consider stage, data preparation is facilitated using the augmented BI platform and capabilities, which make data processing easier and more automated. In the Analyze stage, automated analytics is performed along with AutoML algorithms to generate insights. Finally, in the Interpret stage, data storytelling using NLG uncovers hidden data insights [9].

### 2.1. Generations of BI

There have been three significant generations of BI, which are identified as BI 1.0, BI 2.0, and BI 3.0.

#### 2.1.1. BI 1.0

BI 1.0 was essentially an OLAP-based solution, associated with Enterprise Data Warehouses (EDWs), management information systems, executive information systems, and decision support systems. The technologies used during this first BI generation utilized simple statistical techniques. The reporting had simple capabilities with no ad hoc reporting and usually took time to generate basic reports [10]. For example, when a static report was required, the IT staff would run queries on behalf of business users.

#### 2.1.2. BI 2.0

BI 2.0 introduced analytics as part of BI, with greater expansion of data warehousing, enhancements to EDW, and data integration. Moreover, it introduced the concept of self-service BI analytics, with which users could perform many BI tasks and build their own Key Performance Indicators (KPIs) by using ad hoc querying. Even non-technical users were able to prepare data for analysis, shortening the time it took to gain insights, and reducing IT bottlenecks [10]. For instance, business users could use data discovery and visualization tools in BI to perform analysis.

#### 2.1.3. BI 3.0

BI 3.0 is primarily an app-centric strategy, as seen by its collaborative aspect, where it can be used anywhere, at any time, and on any device or platform. This BI generation includes the AA features in the business analytics cycle. It is built on social workgroups and allows users to create, deliver, analyze, and manage material independently. Moreover, it allows decision-makers in organizations to create and examine content speedily and simultaneously on their own. Furthermore, it uses the power of AI combined with BI. The decision-making process has become more automated with the use of ML, NLP, NLG, and many technologies that facilitate analytical activities [10]. In this era of competition, data have become valuable assets for all businesses, and decision processes are becoming more data-driven. In the revolution of Industry 4.0, large volumes of data are processed at great speed because of the increase in the use of IoT sensors, cybersecurity applications, cloud platforms, and many others [11]. Thus, to ensure that data can be converted to valuable insights, AA is needed to facilitate the analytical cycle by automating many steps and reducing manual work. As such, the analyst will be able to save time, automate most of the analysis tasks, and share more useful insights.

### 2.2. Reasons for Expanding to AA (BI 3.0)

Some business firms, companies, and organizations have converted to using AA because of the many advantages that it can provide for business. It helps businesses to understand the relationships between associated factors. Extracting relevant insights with traditional BI platforms and self-service BI has become increasingly difficult as data complexity has increased. In addition, it makes businesses prone to choosing between sacrificing the quality of their insights by limiting the analytics to a small number of variables, or devoting a significant amount of time to data preparation, analysis, and modelling in order to obtain the in-depth, granular data required. Current BI platforms require manual data preparation and the organization of data, which is not only time-consuming but also inefficient and prone to human bias, since the quality of information is determined by queries made by business users. Thus, users may miss hidden patterns, resulting in missed opportunities.

However, AA automates data preparation and supports the generation of insights with more accurate results than the current self-service BI. Augmented BI platforms can clean, merge, and transform data from different platforms quickly. Many people use AA technology in their organizations, making it possible to meet customers’ needs. AA enables leaders to make data-driven decisions. It also reduces the cost of cleaning and reporting data because it utilizes AI and ML to computerize many processes. Furthermore, AA platforms solve complex business-related problems and help accelerate decision-making for managers and improve the quality of decisions [3]. Table 1 shows some of the expanded capabilities of AA compared to BI [8].

### 2.3. How AA Platforms Are Used in the Business Analytics Cycle

The analytic cycle’s stages (i.e., data preparation, data discovery, model development, and deployment) take advantage of AA to automate most of its tasks. Data preparation is a time-consuming task that requires considerable effort from analysts [6,8]; automating this stage would save time for analysts and allow them to focus on adding value to other stages of the analytics cycle. Using AI helps to achieve smoother data cleaning and preparation tools, such as Decooda’s AI-based engine [12], which can help in preparing and reorganizing, cleaning and outlier filtering, and transforming data using automated processes. Moreover, IBM’s InfoSphere Advanced Data Preparation [13] can provide recommendations for data preparation by using ML via a dashboard that extracts variables that have a high correlation with one another. In data analysis and visualization, many powerful tools are used to execute the processes, such as Qlik Technologies [5] and Tableau [14] platforms that use AI-based capabilities to recommend visualization types based on the input data and to find correlations and relationships between variables. For model development, the AA platform tests and ranks ML algorithms and model parameters based on their ability to enhance the productivity of modelling and reduce the risk of biases in model selection. Some such AA platforms that support Automated Machine Learning (AutoML) include DataRobot [15], H2O.ai [16], and Anodot [17]. In addition, Anodot uses ML to monitor the complete process from development to deployment and to control poor releases that could affect performance [17]. As augmented BI solutions may leverage AI to automate most stages in the analytics cycle, it is not yet feasible to eliminate human involvement, especially in the first and last phases of business problem or opportunity identification, decision-making, and action-taking [6]. The level of technological maturity of algorithms is insufficient to delegate accountability and data governance to them; however, tools such as SAP’s Smart Discovery can be used to assist in these stages by providing recommendations, providing data insights using NLG, and answering queries using NLP [18].

The most popular platforms that leverage AA features, according to Gartner [19], are shown in Table 2.

## 3. Related Work

This paper reviews recent studies from 2018 to 2021 that are related to the benefits of utilizing augmented BI platforms to enhance the analysis of business activities. AA has been proven to handle data effectively in many industries, including healthcare, finance, logistics and transportation, and manufacturing.

### 3.1. Benefits of Utilizing Augmented BI Platforms

A previous study [22] identified the perceptions of Romanian companies about the advantages and limitations of augmented BI solutions and the impacts of these tools on managerial decisions and business evolution. The statistics showed that augmented BI helped respondents to obtain faster and more accurate reporting, save time, improve strategies and plans, improve tactical decisions, implement more efficient processes, enhance customer service, save costs, improve decision-making, and increase revenue. Despite the fact that implementing augmented BI is not an easy milestone, senior executives believed that it was an important step in order to achieve a competitive advantage, and they claimed the need to build effective communication, a culture of change, and proper infrastructure, and to transform decision-making activity to become more information-driven. The capabilities of AA can improve BI platforms by adding new features and attracting more companies to adopt the tool. The research in [23] focused on providing sales insights and forecasts to help the company’s sales strategy. Oracle was used for data preparation, analysis, and visualization, creating models using ML, and using AA with natural language queries to obtain useful insights. The researchers believed that the augmented, integrated, and collaborative features brought many benefits, especially when they were used in the cloud rather than on the premises. The next step would be leveraging the capabilities of DL and AI to build models using autonomous databases and allowing users to obtain answers through the processing of the queries that they type. Another study [24] discussed the state of Moscow’s social sphere by relying on an open data portal and using the power of augmented BI to identify key factors that affected the metrics of the study and clustering of the Moscow region. The ML capability in Power BI helped to perform deep analysis and to automatically search the data for interesting patterns, in order to interpret and rank factors of influence from the most to the least significant. NLP was also utilized through an interactive “Q&A” function in Power BI that recognizes question words and finds answers from the data through visualization that automatically updates as the question changes. These technologies help uncover hidden patterns for users to make informed management decisions.

### 3.2. Healthcare Industry

In the healthcare sector, augmented personalized healthcare helps to improve healthcare services and continuously monitor patients’ physical, personal, social, and medical data by using AI-enabled analytics. One study [1] claimed that augmented BI helps in using available data for disease diagnosis, recommending potential treatments faster and more effectively, and improving drug development and manufacturing. Another study [25] found that augmented BI platforms help to improve the quality of services in the healthcare sector as they save time by helping doctors and specialists to track information easily. Leading augmented platforms have been technically analyzed in terms of applicability, general characteristics, and standard requirements for healthcare tasks. These platforms have been found to reduce costs in the long term and to offer several capabilities that help specialists to track electronic health records and information efficiently, thereby allowing them to concentrate more on patients and giving them extra time.

### 3.3. Financial Industry

The research in [1] examined the role of AA in banking using quantitative text mining with qualitative content analysis for different cases in academic and practical publications. The results revealed that the use of AA in banks, financial services, and insurance results in more accurate forecasts, decreases costs, increases revenue, improves fraud detection, and mitigates risks by allowing continuous auditing and monitoring. It also helps in promoting marketing strategies and customer loyalty policies, as it leads customers to gain trust and invest more. Moreover, the research in [26] highlighted the importance of finance leaders keeping up to date with tools that provide real-time data processing for the agility and efficiency of their organizational processes and for helping their businesses to grow. The main objective was to emphasize that companies need to transform the ways in which they use their data in order to compete in the global market and make their processes more efficient. The research highlighted that financial analysis is more efficient when all sources of data are integrated and visualized for easy understanding. Tableau, which is a popular augmented BI tool, was used in this case and helped users with no programming skills to easily connect, clean, and visualize data and to apply the required analytics more effectively. Furthermore, a qualitative study [27] addressed the implications of big data analytics on financial reporting in different industries. Companies in different industries, including aviation and telecommunications, require a large volume of data processing in their billing systems, requiring big data analytics for revenue recognition. Accordingly, accountants consider big data analytics as an opportunity to work on forensic and valuation areas. The study concluded that the big data ecosystem and new financial technologies would maintain and enhance the company’s competitive advantages.

### 3.4. Logistics and Transportation Industry

AA is also effective in the transportation and logistics sector. The research in [1] claimed that AA enables the digital supply chain by continuously analyzing data and automating decision-making with optimized insights. Some of the supported services that are related to transportation include forecasting incidents and optimizing predictions using historical vehicle delays. Moreover, a study in [28] focused on solving a logistical business challenges by enhancing the analysis of data and visualization by applying augmented BI to help in reporting for supply chain managers. The goal was to optimize cargo spending for inbound and outbound shipments. The study used qualitative research methods and recommended an improvement to the current process by building a data warehouse and then using the Power Query and Power BI tools to generate a freight data dashboard with specified metrics. As a result, new trends and stories were identified from transportation data that helped key users from the logistics team to overcome problems and reduced the costs of the freight operations. Another study [29], using industry data, illustrated that investment in analytics leads to the generation of higher ROI, revenue, and profit than competitors. Thus, augmented BI and predictive analytics support companies in achieving a competitive advantage. For examples of cases in the aviation industry, predictive maintenance tools in the cloud have been developed by Airbus (“Skywise”) and Delta Airlines (“Predictive Maintenance Services”). These tools, which utilize DS and ML techniques, have helped companies to reduce delays, improve the accuracy of prescribed maintenance actions, and prevent accidents, thus protecting passengers.

### 3.5. Manufacturing Industry

AA is also helpful in manufacturing operations. The research in [30] focused on the concept of storytelling with data for the shop floor of a chemical industry company—a manufacturing cell, in particular—to generate a real-time story to simplify the interpretation of the data by utilizing human visual and cognitive abilities. The data story was visualized on a dashboard created in Power BI, and it showed an informative overview of the activities and operations of the cell, with useful insights. As a result, data visualization using the dashboard and storytelling helped in real time in the case of errors or discrepancies that might be encountered on the shop floor along with processes. Furthermore, [31] analyzed company status reports from current control systems to determine alarm limit settings. ML was utilized for alarm classification, and a dashboard was created in Power BI to visualize an optimized overview of the plant process. The results showed that introducing safety modules for automated control systems with the use of data combined with human experience would lead to improved decision-making and eliminate human errors.

It should be noted that researchers in the previous studies focused on the power of augmented BI platforms to make the most of data and improve processes in different industries. However, to the best of our knowledge, there has been no study that demonstrates how AA capabilities have enhanced the traditional BI solutions in terms of time, accuracy, easiness, and other factors that may affect the business analytics process. Therefore, this paper presents a comparative study of the two approaches—particularly in the retail industry—and shows how AI is transforming BI to keep pace with complex data by utilizing AA.

## 4. Proposed Method

This study aims to demonstrate how AA can facilitate the modern analytics cycle compared to traditional BI approaches. Data analysis was performed using both approaches, and their performance was compared by following the phases of the Cross-Industry Standard Process for Data Mining (CRISP-DM) [32]. To reduce bias, the traditional BI approach was performed first, followed by utilizing the AA capabilities, including AI-generated insights, automated and accelerated analytics creation and data preparation, search and conversational analytics using natural language interaction, and ML and predictive analytics.

The dataset used in this study was obtained from a private Saudi retailing company with their consent for its use in this study. The dataset contains 26 original attributes and 170,939 records with no missing values. RapidMiner was used in the traditional approach with Microsoft Power BI Desktop, while the augmented BI platform Qlik Sense was employed for the AA approach. Many platforms support AA; however, Qlik was selected, as it is considered a leader in the Gartner Magic Quadrant for analytics and AI-fueled BI platforms as of 2021 [19]. It offers many extensions that support the analysis process and provides a quick way for users of any skill level to use the power of data to uncover insights. Insight Advisor acts as the intelligent AI assistant in Qlik Sense. It generates advanced analyses and insights automatically, helps prepare data for analytics, and supports conversational analytics and other forms of natural language interaction. Qlik AutoML was employed to generate the Machine Learning models used in this study. It identified key drivers in the used dataset and built the ML models. It also allowed us to refine the models to make predictions and test “what-if” scenarios.

**Figure 1:**
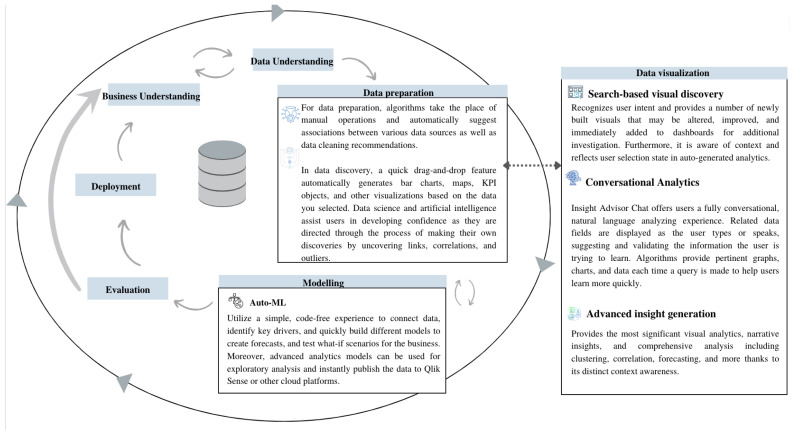
The general structure of the proposed approach.

The general framework of the proposed approach is described in Figure 1. The structure of the proposed approach was inspired by the Cross-Industry Standard Process for Data Mining (CRISP-DM) [32] together with Qlik Sense platform. The data mining process (CRISP-DM) was followed in this work, along with utilizing the AI capabilities in the Qlik Sense platform for each phase.

### 4.1. Business Understanding

The company operates in a highly competitive market, and its business model focuses on providing grocery shopping and delivery services through peer-to-peer mobile applications. The company aims to manage the performance of the agents who deliver orders for their customers. The key metrics, in this case, are agent retention costs, attendance measure, churn rate, and other factors that can help the company to measure its overall performance and optimize the performance of the agents. This phase is frequently considered to be outside the scope of BI and analytics tools and is currently difficult to automate using AI; thus, it was performed manually.

### 4.2. Data Understanding

**Figure 2:**
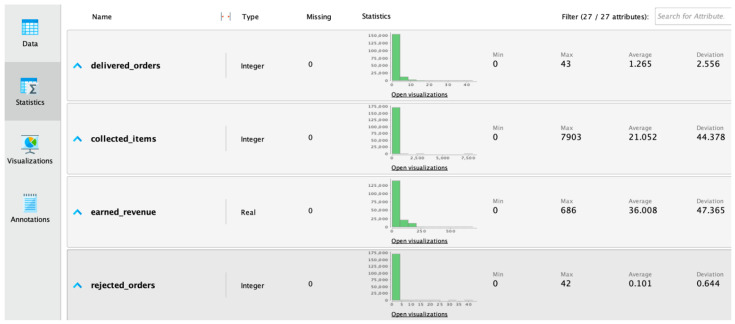
Snapshot of the statistics summary for the traditional approach using RapidMiner.

**Figure 3:**
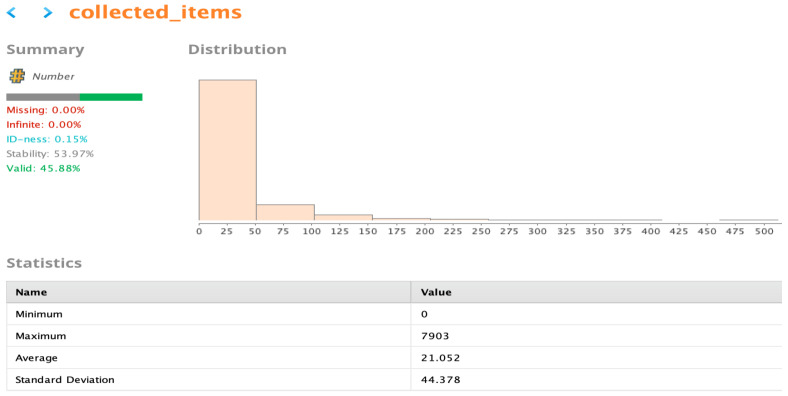
Attribute statistics detail for the traditional approach using RapidMiner.

The statistical analysis was carried out using RapidMiner to explore the data and to look at the variable characteristics. This was performed by connecting the statistics operator on the application in order to view the statistical results. All of the attributes were presented in the statistics page summary, as shown in Figure 2, and the operator “statistics” was used to obtain detailed descriptive statistics of every attribute, as shown in Figure 3.

**Figure 4:**
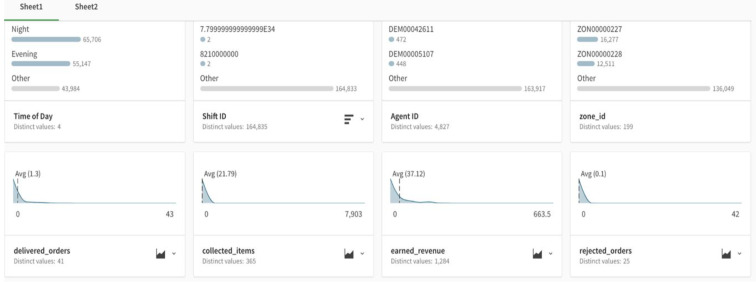
Snapshot of auto-generated data profiling in the AA approach.

Using AA capabilities, the platforms offer intelligent profiling that automatically processes data types and shows descriptive statistics. In this case, Qlik Sense was used, and data profiling that showed all attributes on one screen was generated with one click, as shown in Figure 4. This step is helpful to easily identify null values, distribution, inconsistent values, and outliers.

In this stage, the traditional approach and AA were somewhat similar in performance, as both produced a summary of attributes to enable a better understanding of the variables. However, AA was slightly faster, as it displayed the results in 3 s while RapidMiner took 10 s. Moreover, AA shows the visual summary of all fields, as opposed to RapidMiner, which shows every field separately.

### 4.3. Data Preparation

**Figure 5:**
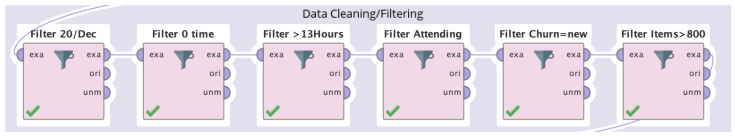
Snapshot of the data transformation process for the traditional approach using RapidMiner.

For traditional analytics, different operators in RapidMiner were connected and used for this phase (such as map, filter, and select attributes). A snapshot of the operators that were used in transformation is shown in Figure 5. It should be noted that traditional platforms—in this case, RapidMiner—provide basic data preparation capabilities and require running the process every time to see the changes and determine whether to proceed with it or apply some manipulations.

However, AA automates this activity by providing association recommendations to easily combine different data sources, and by utilizing ML to detect data relationships, correlations, and advise the best strategy and execute data modelling. With the use of visual drag-and-drop features, it became easier for us to visually integrate, transform, and load data from different sources and to determine the most suitable associations to link them together. Qlik Sense provides simple and easy-to-use functions that help transform data and replace values.

**Figure 6:**
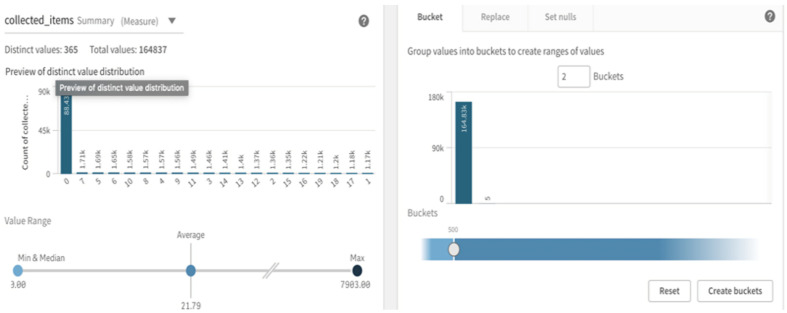
Data preparation screen for the Qlik Sense platform with the AA approach.

**Figure 7:**
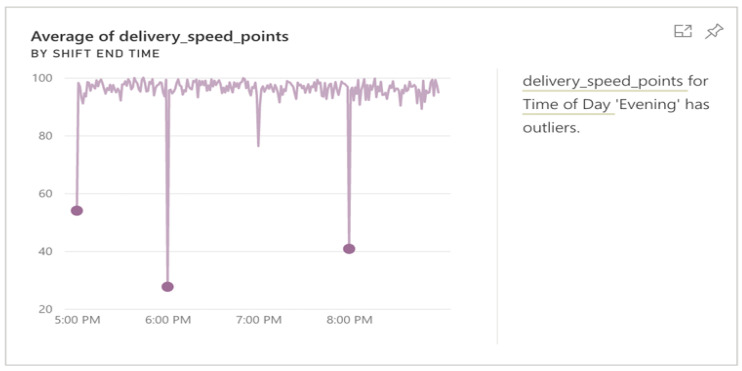
Outlier detection using quick insights.

Qlik Sense also shows descriptive statistics for the field being processed and provides a general overview alongside the transformation window. In this case, it can be easily noted from Figure 6 that there is an outlier, as the average is 21.79 and the largest value is 7903. Qlik Sense also provides a feature for quick insights about the data before starting the analysis. This step is helpful, as it gives us familiarity with important attributes and helps in detecting obvious outliers, as shown in Figure 7.

When we compared the two approaches, data transformation was easier with the AA approach, as the results were reflected immediately. This allows users to see changes and apply further preparations. Furthermore, AA offers faster processing, as each step is performed independently, whereas the traditional approach requires running the complete process every time to see the results. This can be considered time-consuming when the data volume is large.

## 5. Modelling and Evaluation

### 5.1. ML Models

As the company’s revenue model is based on delivering customer orders, the agents who deliver these orders are considered important assets for the company. Therefore, it is necessary to improve agent retention by reducing churn to increase the company’s profit and decrease costs over time. This guarantees that the company will reduce costs when it acquires new agents and will maintain a good relationship with current agents by increasing their retention rate. Thus, predictive models can be built for churn cases to help maximize business value by considering these cases and providing services for them to increase their retention and form any campaigns required.

**Figure 8:**
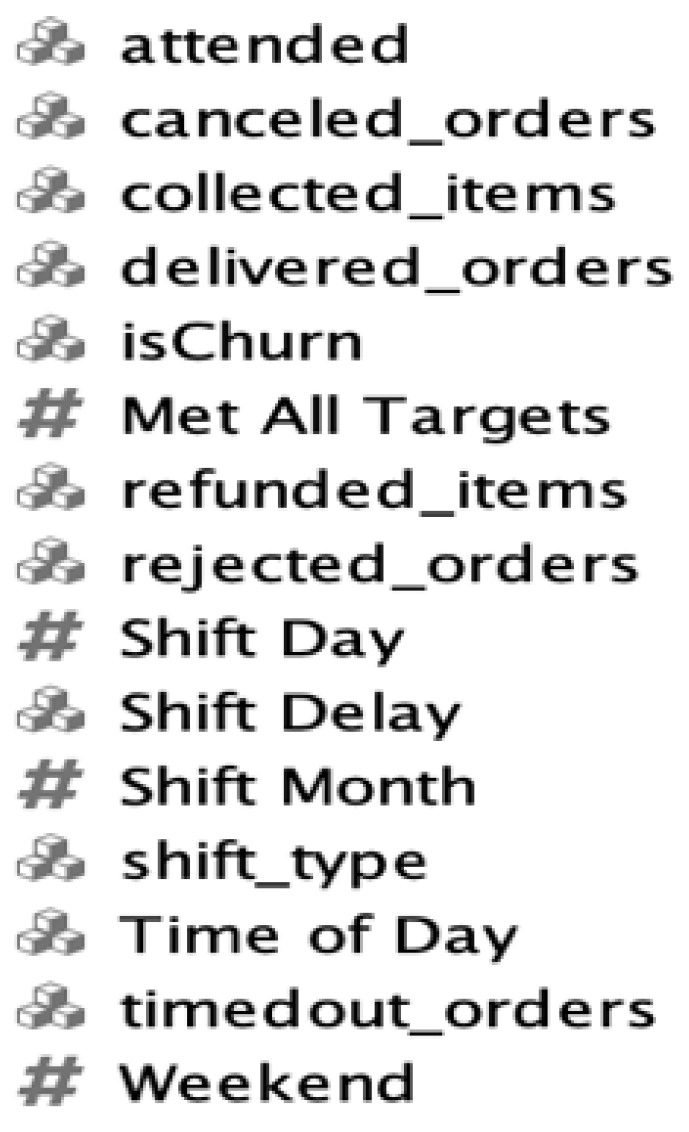
Sample of important selected variables.

After the data were prepared, the most important variables were selected for classification modelling, as shown in Figure 8, and the target label was (“Is Churn” with 0 = returning, 1 = lost). An undersampling technique is employed to balance the data, as the target prediction class was a minority class.

ML algorithms are helpful in selecting the attributes, as they automatically disable inapplicable fields that have high cardinality, such as Shift ID, and apply impact encoding for attributes with large values, such as Zone ID. In addition, the level of importance for each attribute is shown to make it easier for the selection process.

For the traditional approach, different ML models—including Decision Tree (DT), Random Forest (RF), Naïve Bayes (NB), and Logistic Regression (LR) models—were tested to obtain optimal results with the use of cross-validation to mitigate the effects of overfitting. Every model was tested several times by tuning the parameters. Each iteration took approximately 3 min to execute, and the results were noted as shown in Table 3. After sufficient analysis, the RF model was chosen as the best model to predict churn cases, considering different measurements (accuracy, recall, and precision were 70.81%, 77.98%, and 63.88%, respectively). For churn prediction, we believe that the recall is important for preventing as much churn as possible to reduce the company’s costs. Thus, deploying a model with a high recall ensures that the company knows which agents are likely to churn, and this allows the company to react ahead of time.

**Figure 9:**
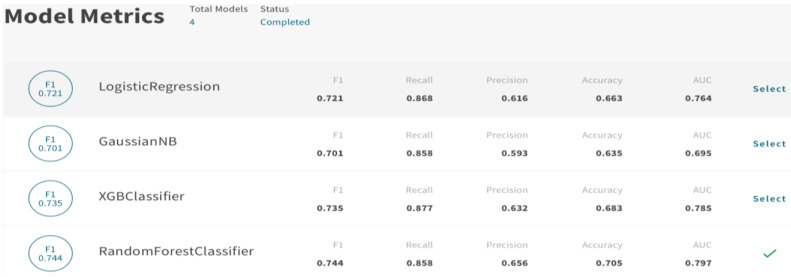
Summary of the generated models’ performance with the AA approach using the Qlik Sense platform.

On the other hand, Qlik Sense AutoML technology allows us to use advanced AA capabilities, including predictive analytics and “what-if” scenario planning, without having to write code and deploy models using an API. This future was compared with the traditional BI approach for analysis. It was found that AutoML supports model building over a short time and allows us to easily adjust the variable selection and the parameters, and to refine the analysis until the best results are scored. Different ML models (i.e., RF, LR, NB, and XGBoost) were deployed in this platform. A summary results of the models’ performance appeared rapidly in the platform (around 1 min), as shown in Figure 9. Furthermore, the platform shows all aspects related to the analysis that are important on a single screen; this includes the correlation of attributes, model status, feature importance, and SHapley Additive exPlanations (SHAP) values, in order to give us recommendations to improve the model if required or show the strength of the analysis. Moreover, we can take advantage of “what-if” analysis by using the scenario feature that enables changing the values of attributes and obtaining a prediction accordingly. This helps with numeric values, such as changing the expected numbers of delivered orders, refunded items, or any other variable, and predicting the case and churn status.

After refining the models and conducting several tests, it can be seen from Figure 9 that the RF model achieved the best results among the other classifiers. Thus, it can be seen that RF is the best performing model for the used dataset in both approaches. Humans can work as effectively as automated platforms; however, utilizing the AA capabilities of AutoML can enhance human-centric analysis, save time, empower the analysis process, and allow the user to evaluate the model on a dataset and obtain predictions for future data.

To summarize, Qlik Sense AutoML helps in finding the best parameters and generating automatic insights that can be beneficial in decision-making. The advanced features of AA algorithms helped the analytics and enabled the business to make predictions of agents’ churn to minimize costs that the company might face. Having a model with high recall will ensure that the business knows the agents who are likely to churn, enabling them to react in advance. The model is ready for the business and can be fed with new data whenever needed to identify the returning agents and give them compensation to avoid losing them. Thus, we can say that ML algorithms helped in providing insights into the current situation and the future prediction to find what can be done for enhancements.

### 5.2. Data Visualization

To dive deep into the dataset and to find further insights, important attributes that may help in decision-making were visualized. This step usually requires a significant amount of time to identify useful measures, interesting patterns, and relationships. Following the traditional analytical approach, a dashboard was built using Microsoft Power BI Desktop. The BI platform helped in selecting different attributes and choosing a suitable visualization for the user. The design phase took considerable time, as the process began with identifying the most important relationships and then trying to visualize them and determine whether they would be useful, followed by deriving insights from them to help decision-makers to obtain value.

**Figure 10:**
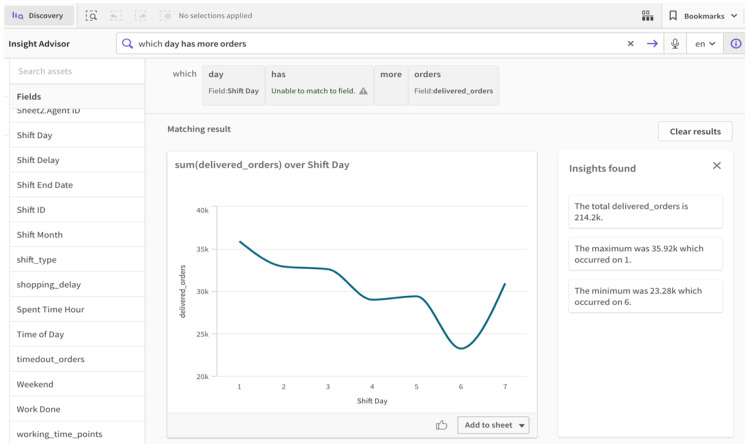
Natural language understanding of the user’s query in Qlik Sense with the AA approach.

Nevertheless, the AA capabilities provided in BI platforms can accelerate the analytics and visualization process and reduce the risk of missing important insights. The augmented features of Qlik Sense were used in this case to facilitate the design of the dashboard. Insight Advisor was used to automatically generate quick insights and learn the important relationships suggested by the system. Although not all of them were useful, it was helpful to receive quick hints on important attributes to explore and obtain further analysis. The platform also allows for selecting any attribute and then automatically suggesting a list of visualizations with suitable types based on the data. Furthermore, the platform allows us to ask questions and to obtain quick answers, as it supports natural language interaction for conversational and search-based analysis. NLP was used to ask the question “Which day has more orders?”, and the results were displayed both visually and in a form of narrated natural language (i.e., NLG) from the derived insights, as shown in Figure 10. It can be seen from the results in Figure 10 that Friday has the lowest number of orders delivered by agents; thus, this creates another question as to whether the reason was due to the few orders processed by customers, or because few agents were working on that day. Consequently, this provides insights for decision-makers to give offers on that day in order to retain agents and to obtain more orders from customers, leading to increased profits.

**Figure 11:**
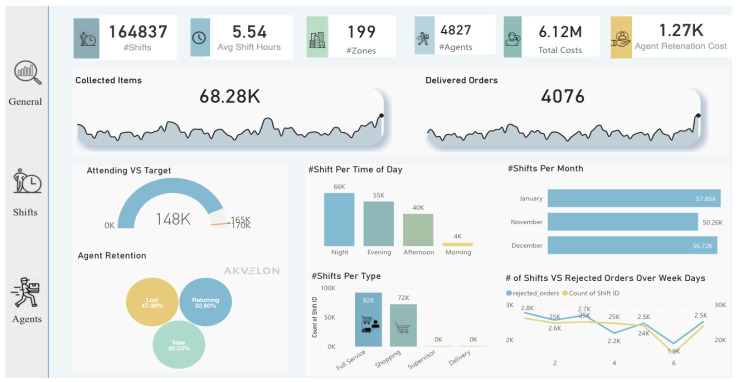
Dashboard (general tab) designed after analysis.

**Figure 12:**
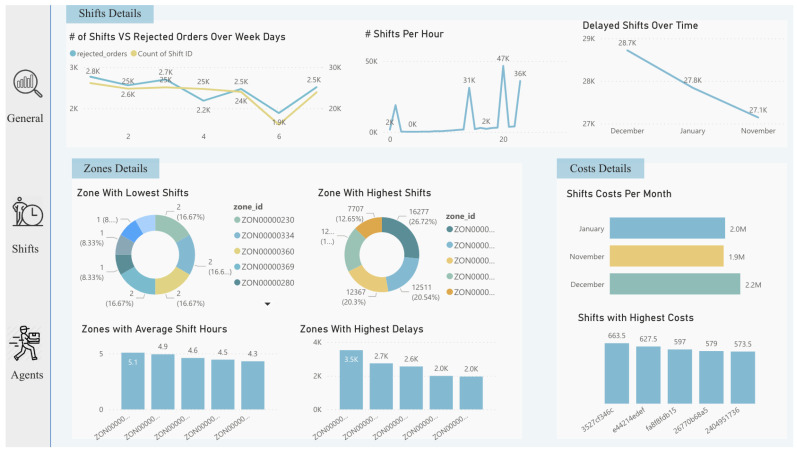
Dashboard (shifts tab) designed after analysis.

**Figure 13:**
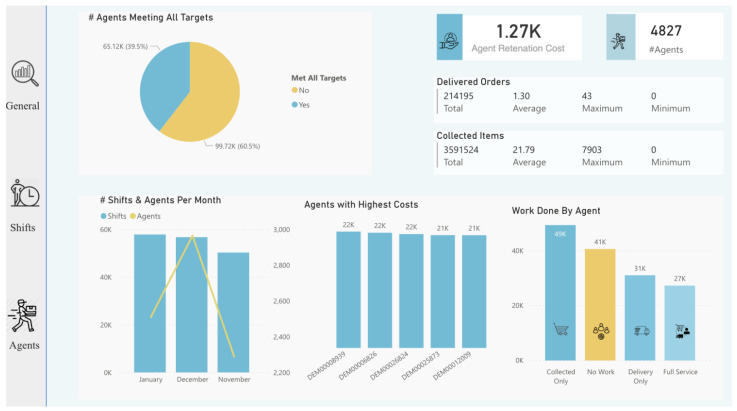
Dashboard (agents tab) designed after analysis.

**Figure 14:**
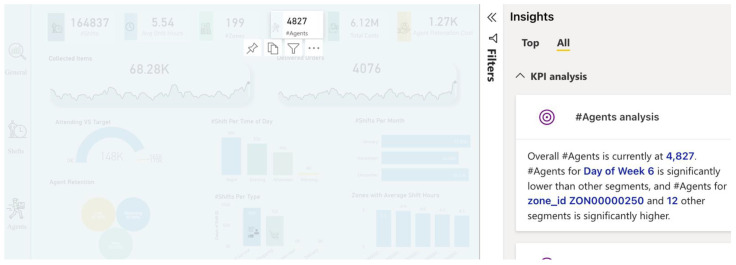
Auto-generated insights from the dashboard in the AA approach.

Lastly, after diving into the analysis process and taking advantage of the features provided by AA, we imported all useful visualizations into the dashboard to deliver insights for decision-makers, as shown in Figure 11, Figure 12 and Figure 13. Some insights could be automatically generated, as shown in Figure 14, by clicking on the “get insights” feature, while other insights needed to be identified by critical thinking and drill-down by decision-makers. Such actionable recommendations focus on re-engaging the agents to reduce churn, because the churn rate was identified as high, with 47% of agents “Lost”. This can be accomplished by implementing an agent rating system, assessing agent feedback, awarding returning agents with incentives and discounts, and providing first-time agents with guidance on how to perform effectively during shifts. Furthermore, many shift bookings went unattended by agents; thus, the company should investigate the shift scheduling system and consider imposing fines on agents who booked shifts but did not show up twice or more. Moreover, many shifts were registered with no work done by agents, indicating the importance of introducing an agent work schedule to manage the shift work. Furthermore, many agents failed to reach the company’s targets (i.e., working time and delivery speed); this shows that the target setting needs to be reconfigured, and performance managers must determine the underlying reasons. Finally, in certain zones there are few shifts (i.e., just one or two), and the marketing team should attempt to develop marketing campaigns in these locations.

Hence, it is important to mention that AA only facilitates the process, but the main workload rests on the analyst, as they are responsible for performing all of the analysis, getting the most out of the data, and building an effective dashboard. Based on the dashboard, decision-makers will be able to derive actionable insights and improve business performance.

### 5.3. Deployment

The recommended model for deployment is the RF model. This model is ready to be deployed for the business and can be automatically retrained with fresh data and redeployed whenever necessary to optimize the agents’ overall performance and focus on returning agents. The AA helped in completing the analysis cycle more smoothly as it simplified the data preparation process, supported the generation of automatic insights, and saved time. The company benefited from the proposed model as it helped in giving insights and future prediction of the company’s agent situation, since the behavior of the agent has a significant impact on customer satisfaction and the bottom line. Agents are critical elements of the company business model, which is why the business needs to constantly monitor their performance and develop appropriate strategies to alleviate any customer loss.

## 6. Discussion

It can be seen when comparing both approaches in the analysis process that AA simplifies the traditional analytics process by automating many steps, as shown in Table 4. Traditional BI facilitates the analytics cycle for businesses by providing drag-and-drop features that enable the preparation and visualization of the data. We can say that traditional BI is useful for analyzing data of small-to-medium scale, but it will be challenged by big data analysis. However, AA provides additional AI features to traditional BI platforms that enable many functions to be performed with one click. AA uses the techniques of ML, NLP, and NLG to easily sift through the data and find correlations and useful insights. By integrating AI into the data analysis process, businesses may automatically clean, assess, and visualize their data. In addition, AA helps in automating ML algorithms for analytics users and teams to easily generate models for business, make predictions to understand potential outcomes, and test business scenarios. Furthermore, it supports conversational analytics with the help of NLP and NLG to ask questions and receive answers.

Based on experiencing both analytical approaches, we can say that AA helps facilitate the analysis process but does not eliminate the need for the analyst’s skills in the analysis. AA is useful for automatically generating insights; however, not all insights are useful to the business goal of the analysis. Some of the insights were used in further analysis and facilitated the discovery of interesting relationships among attributes.

In terms of time consumed, the analytics of the traditional BI approach took much more time than AA, particularly in model tuning, as the process took 3 min for every model to generate results, whereas AA generated all models within 1 min.

For dashboard design in AA, some insights were automatically generated, whereas other insights had to be identified by decision-makers. Thus, we can say that AA is helpful in analysis to a certain point, but we cannot rely on it completely to perform all of the analysis and to generate insights, as decision-makers play an important role in sharing and operationalizing findings.

## 7. Conclusions and Future Work

Businesses should always utilize the oil of the digital economy—the data—as much as possible in order to succeed in the market and gain a competitive advantage. Analytics and BI are key facilitators in extracting value from business operations, and they support and facilitate better business decisions. However, extracting relevant insights with traditional BI platforms and self-service BI has become increasingly difficult as data complexity has increased, especially with the introduction of big data analytics. As the next generation of BI (BI 3.0), AA enhances the self-service BI model in many ways with the use of AI. AA utilizes AI algorithms to facilitate data preparation and to suggest associations and cleaning tasks. It provides AutoML algorithms to generate insights and make predictions. It also incorporates conversation analysis with NLP by allowing users to ask questions and using NLG for delivering insights and data storytelling. In short, AA features enable data analysts and citizen data scientists to build custom visualizations, boost modelling accuracy, and generate insights in a simple and timely manner.

This paper addresses a comparative study between the capabilities of traditional BI and its enhanced AA platforms in the business analytics cycle. It covers the generations of BI, the reasons for expanding to AA, how AA platforms are used in the business analytics cycle, and the benefits of utilizing augmented BI platforms in different industries. Moreover, data were tested with both approaches to find out how AA futures enhance the BI analysis process. The results revealed that AA accelerates the analysis process and reduces the time it takes to obtain valuable results. Moreover, it boosts the accuracy of ML models and automates model tuning. Furthermore, it supports visualizing the data to find hidden and useful insights. In general, AA enhances the BI platform and makes it easier to use. It has become more valuable for the average business user, thus increasing data literacy. However, AA does not eliminate the use of the analyst’s skills in identifying actionable insights, as not all automatically generated insights are useful.

Since the research in AA is limited in the literature, further studies could address the concept of an “augmented consumer”, which delivers new augmented user experiences in BI and analytics platforms and expands the process from analyst-focused to consumer-focused.

## Figures and Tables

**Table 1:** Expanded features of AA compared to features of classical BI.

BI	AA
OLAP	Search-based visual analysis and conversational analytics that is NL-driven (NLP)
Ad hoc queries	Interactive and informative analytical dashboards
Dashboards and scorecards	Data storytelling
Reporting by tracking KPIs and metrics	Predictive and prescriptive analytics
Descriptive analytics	Real-time advanced analytics
Operational and real-time BI	Scenario analysis
Manually preparing data and digging deeper into the data for more targeted information	Smart discovery and automated insights
	Accelerated data preparation
	Big data analytics

**Table 2:** Popular platforms that leverage AA features.

Tool	Description
Qlik	Qlik’s Sense is a high-performance tool that allows users with different analytical levels to search and analyze any dataset. Qlik’s AA component, Insight Advisor, facilitates data exploration by automatically generating insights based on data analysis, which automates and speeds up the data preparation process. Its search-based visual analysis displays hidden insights as powerful visuals that can be modified and adjusted to create effective dashboards. Furthermore, NLP is used in conversational analytics, which allows users to evaluate data in a conversational manner [5].
Power BI	Power BI allows analysts to perform data preparation, data discovery, and building of dashboards using similar design techniques. The platform works with Excel and Office 365 and consists of an active user community that builds the tool’s potential. Power BI’s analytical capabilities are enhanced by the availability of powerful AA capabilities and ML algorithms. Furthermore, features such as Quick Insights and Q&A visualizations allow users to easily examine and interpret data. Other elements, such as text analytics and visual analytics, enable customers to successfully employ the analytics capabilities in their data analysis [20].
Tableau	Tableau fully integrates Einstein analytics to leverage AI technologies to examine and analyze data in order to make predictions and recommendations based on those findings. The presence of features such as Ask Data and Explain Data demonstrates that the industry is moving beyond traditional visualization-based solutions. In addition, Tableau uses smart analytics tools such as NLP and NLG to give customers a better data analysis experience [14].
ThoughtSpot	ThoughtSpot is a BI and analytics company known for its highly scalable and relational analytics search engine, which allows business users to interact easily with data. It is considered one of the first BI suppliers to deliver AI-generated insights throughout the user experience, from a smart homepage to search, dashboards, and datasets. It has a user-friendly interface for providing automated insights and allows users to ask questions and execute queries [21].

**Table 3:** Models’ performance in the traditional approach using RapidMiner.

Model	Accuracy	Precision
DT	65.42%	63.11%
RF	70.81%	63.88%
NB	63.08%	63.93%
LR	64.76%	63.68%

**Table 4:** Comparison between traditional BI and AA approaches in analysis.

	Data Understanding and Reparation	Finding Patterns in Data and Visualizations	Modelling
**BI**	Users perform manual data preparation, cataloguing, and ensure data quality, with limited automated transformation.	Users explore the relationships and patterns in data manually using interactive visualizations, create measures and metrics, and build the dashboard based on how the user interprets the results.	Manually tune models to find the best parameters. Choose a way to cross-validate by running through multiple training and evaluation strategies to obtain optimal results. Manually compare and select between the generated models.
**AA**	AA platform performs automatic data profiling, uses algorithms to recommend data enrichment, finds outliers and correlations, and transforms data via automated processes. AI is used to highlight important attributes useful for analysis.	AI-based capabilities are used to recommend visual types based on the characteristics, correlations, and relationships between variables. Users can utilize conversation analysis by asking questions using NLP. ML helps to generate automatic insights. Insights are narrated and summarized using NLG. Users can perform data storytelling to share insights.	Automatically choose suitable cross-validation techniques, evaluate variable contributions, and select features. AutoML generates and combines models to obtain optimal models and retunes them to obtain optimal results. Rank ML algorithms and model parameters to improve the productivity of modelling and limit the risk of biases towards model selection. AI enables users to perform scenario and “what-if “analysis for future predictions.
